# Proximal Tibia Tumour Location and Curettage Are Major Risk Factors of Local Recurrence in Giant Cell Tumour of Bone

**DOI:** 10.3390/cancers15184664

**Published:** 2023-09-21

**Authors:** Michal Mahdal, Tomáš Tomáš, Vasileios Apostolopoulos, Dagmar Adámková, Peter Múdry, Iva Staniczková Zambo, Lukáš Pazourek

**Affiliations:** 1 First Department of Orthopaedic Surgery, St. Anne’s University Hospital, 60200 Brno, Czech Republic; michal.mahdal@fnusa.cz (M.M.); tomas.tomas@fnusa.cz (T.T.); vasileios.apostolopoulos@fnusa.cz (V.A.); 2 Faculty of Medicine, Masaryk University, 62500 Brno, Czech Republic; dadamkova@mou.cz (D.A.); mudry.peter@fnbrno.cz (P.M.); iva.zambo@fnusa.cz (I.S.Z.); 3 Clinic of Comprehensive Cancer Care, Masaryk Memorial Cancer Institute, 60200 Brno, Czech Republic; 4 Department of Pediatric Oncology, University Hospital Brno, 66263 Brno, Czech Republic; 5 First Department of Pathology, St. Anne’s University Hospital, 60200 Brno, Czech Republic

**Keywords:** GCTB, denosumab, targeted treatment, bone, neoplasia

## Abstract

**Simple Summary:**

The purpose of our study was to examine remaining controversies in key issues about the treatment of GCTB. To achieve that we analysed a cohort of 102 patients treated in our institution between 2006 and 2020. We identified the main risk factors for local recurrence, evaluated the recurrence-free survival in dependence on neoadjuvant denosumab use and the type of procedure, and compared the functional outcomes after curettage and en bloc resection.

**Abstract:**

Giant cell tumour of bone (GCTB) is one of the most common local aggressive tumourous lesions with a wide variety of biological behaviour. However, there are no clear indicative criteria when choosing the type of procedure and the complication rates remain high, especially in terms of local recurrence. The purpose of the study was to (1) identify the main risk factors for local recurrence, (2) evaluate the recurrence-free survival in dependence on neoadjuvant denosumab use and the type of procedure, and (3) compare the functional outcomes after curettage and en bloc resection. The group included 102 patients with GCTB treated between 2006 and 2020. The mean age of patients was 34.4 years (15–79). The follow-up period was 8.32 years (2–16) on average. Local recurrence occurred in 14 patients (29.8%) who underwent curettage and in 5 patients (10.6%) after en bloc resection. Curettage was shown to be a factor in increasing recurrence rates (OR = 3.64 [95% CI: 1.19–11.15]; *p* = 0.023). Tibial location was an independent risk factor for local recurrence regardless of the type of surgery (OR = 3.22 [95% CI: 1.09–9.48]; *p* = 0.026). The recurrence-free survival rate of patients treated with resection and denosumab was higher compared to other treatments at five years postoperatively (*p* = 0.0307). Functional ability and pain as reported by patients at the latest follow-up were superior after curettage compared to resection for upper and lower extremity (mean difference: −4.00 [95% CI: –6.81 to −1.18]; *p* < 0.001 and mean difference: −5.36 [95% CI: −3.74 to −6.97]; *p* < 0.001, respectively). Proximal tibia tumour location and curettage were shown to be major risk factors for local recurrence in GCTB regardless of neoadjuvant denosumab treatment. The recurrence-free survival rate of patients treated with resection and denosumab was higher compared to other treatments. The functional outcome of patients after curettage was better compared to en bloc resection.

## 1. Introduction

Giant cell tumour of bone (GCTB) is one of the most common local aggressive tumourous lesions with a wide variety of biological behaviour [1,2]. Since occurring mainly in young adults and the current knowledge does not provide conclusive treatment approaches, a large discussion regarding the optimal treatment of GCTB has been established. The surgical treatment options are based on the removal of the tumourous mass intralesionally by scraping using a curette (curettage) or extralesionally in en bloc resection [3,4]. However, there are no clear indicative criteria when choosing the type of procedure and most orthopaedic surgeons proceed according to previous experience when dealing with GCTB. As a result, complication rates remain high, especially in terms of local recurrence [5,6].

To reduce the complication rates of GCTB, previous papers have examined the risk factors of local recurrence [7,8,9]. Authors commonly suggested that the female gender, younger age, extraosseous tumourous mass, denosumab use, and curettage are potential risk factors for local recurrence [10]. In a review of ten studies, soft tissue invasion and tumour size larger than 5 cm were shown to be major risk factors [9]. Additionally, a retrospective multicentre study identified that patients treated with curettage achieved higher local recurrence rates compared to those treated with en bloc resection. Finally, there was found dependence between the local recurrence and the tumour location, and proximal fibula GCTB location was a major risk factor [7]. Other described critical anatomical locations include the proximal tibia, radius, or proximal femur [5,11].

The reported rates of postoperative local recurrence for GCTB vary widely, ranging from 3% to 51% [1,12,13]. Although, the reported rates are strongly dependent on the type of procedure and the neoadjuvant use of denosumab [14,15]. Previous studies comparing tumour-free survival in patients depending on the surgical method point out en bloc resection with endoprosthetic reconstruction of the defect to have greater long-term survival and even in cases of pathological fracture [16,17]. The use of denosumab is indicated in cases of inoperable tumour or metastatic disease and for the neoadjuvant treatment of locally advanced tumour (Campanacci grade 3) to enable joint or limb salvage surgery and reduce the incidence of local recurrence [18,19,20]. Recent papers recorded lower mid-term recurrence-free survival in patients treated preoperatively with denosumab [15,21]. Some sources even discourage the use of denosumab when considering an intralesional procedure [22]. Sunitinib, a Platelet-derived growth factor receptor inhibitor (PDGFR), has the potential to be an effective complement to denosumab in the future, causing inhibition of neoplastic stromal cells. As a result, denosumab treatment in combination with sunitinib targets osteoclast-like giant cells and stromal cells at the same time [23].

As described studies revealed that curettage is a major risk factor and achieved lower disease-free rates [7,9,22]. However, extralesional resection is associated with complications in terms of defect restoration and as a result worse functional outcomes [5]. To restore joints after resection, used endoprosthetic tumourous replacements have still high failure rates and those rates increase over time [24]. Taking into account that GCTB predominantly affects young patients the type of procedure should be carefully considered on an individual basis.

Therefore, we examined the main risk factors for local recurrence; the recurrence-free survival of patients depending on neoadjuvant denosumab use and the type of procedure; and the functional outcomes of patients after curettage and en bloc resection.

## 2. Materials and Methods

### 2.1. Sample Characteristics

This retrospective study analysed the records of 102 patients with GCTB treated between 2006 and 2020. The inclusion criteria were patients with GCTB of the girdle and long bones who were surgically treated. As a result, 7 patients were excluded from the study and 95 patients were evaluated postoperatively. These were patients who had an inoperable tumour in the axial skeleton and a diagnosis of primary malignant GCTB. Data was recorded on the patients regarding age, gender, tumour location, Campanacci grade, type of surgery, treatment with denosumab, follow-up and functional outcome (Table 1). Our dataset included 52 males and 43 females with a mean age of 34.4 years (range 15–79 years). The follow-up period was 8.32 years (2–16) on average. En bloc resection was performed in 48 cases, while curettage with local adjuvant occurred in 47 patients, divided into 23 distal femurs, 22 proximal tibias, 18 forearms, 10 feet, 6 proximal femurs, 5 hands, 4 fibulas and 7 other locations. After curettage of GCTB, all cases underwent speed burring, followed by two cycles of phenolisation (80% phenol). Finally, the cavity left after the tumour removal was filled with bone cement. According to the Campanacci classification, the local tumour stage was determined before starting the treatment; in 5 cases it was assessed as grade 1, in 51 cases as grade 2 and 39 cases as grade 3. Pathological fracture occurred in 12 patients and the most common treatment method was wide resection. During follow-up, the disease spread to the lungs in three patients. Treatment with denosumab was indicated in a total of 20 patients, and en bloc resection was performed in 13 of them. Indications for using denosumab included cases of Campanacci grade 3 tumours where surgery would have resulted in significant morbidity and functional impairment. En-bloc resection after denosumab was indicated for tumours that were large in size, with a high risk of incomplete curettage and recurrence. Based on the data obtained, we compared the significance of age, gender, tumour location, Campanacci grade, type of surgery, tumour size, and denosumab administration on the risk of local recurrence (Table 1). Postoperative routine follow-up evaluation was performed every three months for the first two years, every six months for the next three years and then annually. Each follow-up evaluation included clinical examination and imaging methods.

### 2.2. Evaluation of Functional Results

Function was evaluated using the Musculoskeletal Tumour Society (MSTS) scoring system for the upper and lower extremities [25]. This system includes numerical values from 0 to 5 points assigned for each of the following six categories: pain, function, emotional acceptance, hand positioning, dexterity and lifting ability. The values were added, and the functional score was presented as a percentage of the maximum possible score. The MSTS score was assessed at the 2-year follow-up and eventually modified at the last follow-up.

### 2.3. Statistical Analysis

Statistical analysis was conducted using the R software (version 4.0.5) in the RStudio development environment. To answer our first question, Fisher’s exact probability test was used to compare the proportions between the groups. To measure the association between exposure and outcome, an odds ratio was used. To answer our second question, the Kaplan–Meier analysis was used to evaluate recurrence-free survival, and differences in survival between treatment groups were assessed using a log-rank test. To answer our third question, the MSTS score was calculated, and an unpaired t-test was used to compare the functional outcome (MSTS) between the groups.

## 3. Results

### 3.1. Identification of the Main Risk Factors for Local Recurrence

**Figure 1:**
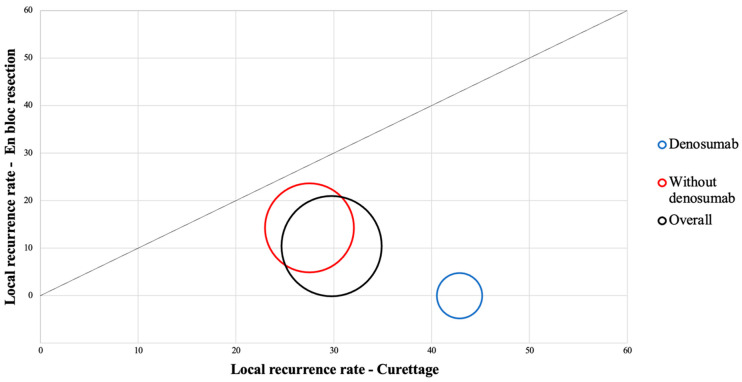
Local recurrence rate chart: Dependence of denosumab use and the type of procedure.

Of the 95 postoperatively evaluated patients, local recurrence occurred in 19 cases (20%). All local recurrences were found at the site of the primary tumour. Local recurrence occurred in 14 patients (29.8%) who underwent curettage and in 5 patients (10.6%) after en bloc resection (Table 2). Curettage was shown to be a factor in increasing recurrence rates (OR = 3.64 [95% CI: 1.19–11.15]; *p* = 0.023). Prior to the procedure, denosumab was used in 20 cases, and local recurrence occurred in 3 cases (15%) (OR = 0.65 [95% CI: 0.16–2.50]; *p* = 0.531). All local recurrences occurred in seven patients treated with curettage after denosumab (OR = 3.37 [95% CI: 0.68–16.58]; *p* = 0.134) (Figure 1). The incidence rate of local recurrence was higher in cases of C3 grade (n = 10; 25%) (OR = 1.80 [95% CI: 0.65–4.95]; *p* = 0.254) compared to C2 (n = 9; 18%) (OR = 0.72 [95% CI: 0.26–1.99]; *p* = 0.537), and no cases of local recurrence were recorded in cases of C1 grade (OR = 0.33 [95% CI: 0.0177–6.2933]; *p* = 0.463). Proximal tibia location was an independent risk factor for local recurrence regardless of the type of surgery (OR = 3.22 [95% CI: 1.09–9.48]; *p* = 0.026) and occurred in eight cases (36.3%). Local recurrences were also found in the forearm (n = 3), the foot and hand (n = 2), the distal femur (n = 2) and other sites (n = 4) (Table 3).

### 3.2. Recurrence-Free Survival Evaluation

**Figure 2:**
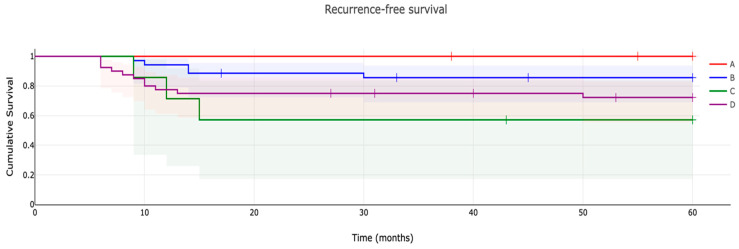
Recurrence-free survival (with 95% confidence interval) of patients with giant-cell tumor of bone: A. Patients treated with denosumab and resection (n = 13). B. Patients treated with resection without denosumab (n = 35). C. Patients treated with denosumab and curettage (n = 7). D. Patients treated with curettage without denosumab (n = 40).

The recurrence-free survival rate (Figure 2) of patients treated with resection and denosumab was 100% at five years postoperatively and higher compared to those of patients treated with denosumab and curettage (57.1%) at five years postoperatively (*p* = 0.0307). The recurrence-free survival rate of patients treated with resection without denosumab was 85.6% and higher compared to those of patients treated with curettage without neoadjuvant denosumab (72.2%) at five years postoperatively (*p* = 0.258).

### 3.3. Functional Outcome Comparison

**Figure 3:**
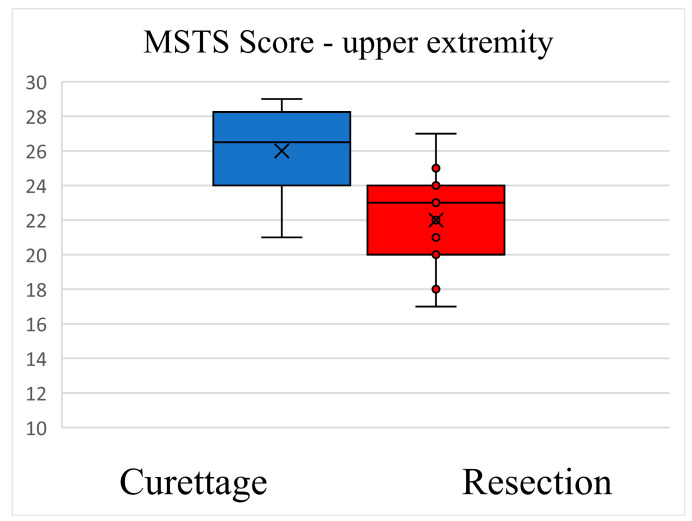
Functional ability and pain score after curettage and en bloc resection—MSTS upper extremity.

**Figure 4:**
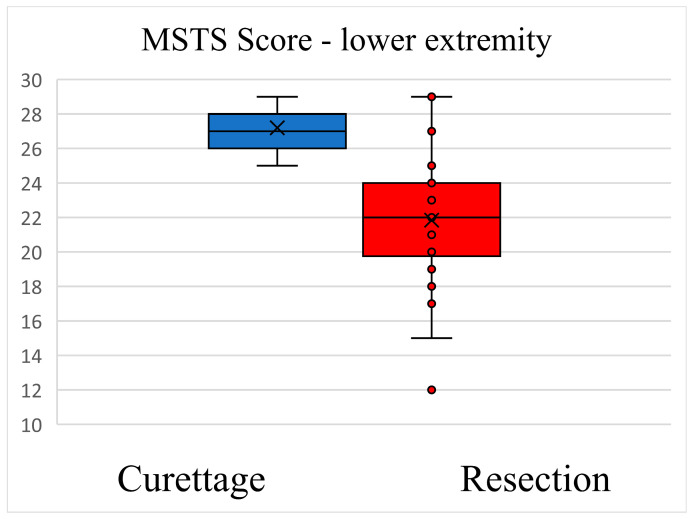
Functional ability and pain score after curettage and en bloc resection—MSTS lower extremity.

Functional ability and pain as reported by patients at the latest follow-up were superior after curettage compared to resection (Figure 3 and Figure 4). The mean MSTS score for the upper extremity after curettage was 26 ± 2.82. The mean MSTS score for the upper extremity after resection was 22 ± 2.77 (mean difference: −4.00 [95% CI: –6.81 to −1.18]; *p* < 0.001). The mean MSTS score for the lower extremity after curettage was 27.2 ± 1.11. The mean MSTS score for the lower extremity after resection was 21.84 ± 3.86 (mean difference: −5.36 [95% CI: −3.74 to −6.97]; *p* < 0.001).

## 4. Discussion

The treatment of GCTB has largely involved surgical intervention, with or without adjuvant therapy [26,27]. There are numerous studies, even multicentric, dealing with this benign disease because of the substantial complications, the frequent revision procedures and the unpleasant results [17]. However, there are still several controversies in key issues about the treatment of GCTB. In particular, there are raised concerns about the increased risk of recurrence of GCTB, especially in patients who received denosumab treatment [28]. Moreover, there is a lack of consensus on the risk factors for recurrence and on how to best optimise treatment strategies to minimize recurrence [19]. Therefore, it is important to conduct further research to better understand the risks factor, considering the patient functional outcome, as well as developing tailored and effective treatment algorithms that take into account the unique characteristics of each GCTB patient.

There are several limitations to this study. First, the retrospective design of the study with the consequent disadvantages. Second, the study was conducted at a single institution, which may limit its external validity. Finally, the study did not consider other factors that may influence the risk of local recurrence or functional outcome such as the use of local adjuvant therapy or cavity fill-up. However, all curettage cases underwent the same adjuvant regime. Despite the limitations, adequate results were obtained.

Of the investigated potential risk factors for local recurrence (gender, age, anatomical location, Campanacci grade, neoadjuvant use of denosumab, tumour size and type of procedure), curettage and proximal tibia tumour location were shown to be major risk factors. In a review study based on 89 articles, curettage for GCTB without neoadjuvant treatment has been reported at a median incidence of local recurrence after simple curettage of 47% but with a high variance (27–82%). A further reduction of the high incidence rate was recorded with the use of local adjuvants [29]. Anatomic location could be associated with the risk of local recurrence of GCTB. Errani et al. described a higher risk of local recurrence of GCTB in the area of the proximal femur with regard to the increasing risk of pathological fracture or osteonecrosis of the femoral head depending on the size of the trepanation hole [30]. Another location associated with a higher risk of local recurrence is the distal radius due to the thin volar and dorsal cortex and the close relationship to the ulna and the surrounding soft tissue structures [31,32]. Like us, Siddiqui et al. mentioned the proximal tibia as a risk site [8]. The reason may be, among other factors, the relatively high risk of complications associated with resection and replacement of the proximal tibia such as infection or soft tissue failure [33]. This fact may lead surgeons to prefer curettage or its combination with denosumab instead of en bloc resection and reconstruction of the defect.

Since 2013, when a new era began in the treatment of GCTB with introduction of denosumab, a number of articles have appeared pointing to a higher risk of local recurrence when combining neoadjuvant denosumab treatment with curettage [14,28,34]. Residual tumour stromal cells may hide within the newly formed cortical rim and thickened cortex and may recur once denosumab is discontinued [34,35,36,37]. Even in our group, the five-year recurrence-free survival after the combination of neoadjuvant denosumab therapy with curettage (57.1%) exceeded the recurrence-free survival after curettage without denosumab (72.2%). However, it should be noted that these two groups differ in the initial extent of the tumour, where denosumab is used to down-stage the tumour, enabling curettage, which would otherwise not be possible, which is also mentioned by other authors [21,38]. In the cases of GCTB Campanaci grade 3, neoadjuvant treatment with denosumab is recommended to minimize the risk of local recurrence and is considered the optimal combination with an en bloc resection procedure. This combination leads to the demarcation of the tumour, facilitates its resection and sometimes even allows a limb salvage procedure [22,39,40]. This fact is also supported by the results of our study where the five-year disease-free survival for resection alone was 85.6% and for the combination with neoadjuvant denosumab therapy it was 100%.

The functional outcome expressed by the MSTS score was higher after curettage than after resection for the upper and lower extremity. Similar results are reported in numerous studies by other authors; generally en bloc resection has been reported to reduce the risk of local recurrence at the cost of worse function [19,30,41]. Downstaging GCTB Campanacci grade 3 with neoadjuvant treatment with denosumab could allow a less invasive procedure (curettage) and ensure a better functional result while simultaneously increasing the risk of local recurrence [16,38,42].

## 5. Conclusions

Considering the limitation, the present study examined the risk factors for local recurrence in GCTB, the recurrence-free survival and the functional outcomes of patients. Proximal tibia tumour location and curettage were shown to be major risk factors for local recurrence in GCTB regardless of neoadjuvant denosumab treatment. The recurrence-free survival rate of patients treated with resection and denosumab was higher compared to other treatments. The functional outcome of patients after curettage was better compared to en bloc resection. The preferred surgical technique in the treatment of GCTB is curettage with the use of local adjuvants due to better functional results, although the risk of local recurrence can be minimised by combining neoadjuvant denosumab treatment with en bloc resection.

## Figures and Tables

**Table 1:** Sample characteristics.

Features	Overall	Resection	Curettage	
Number of patients	95	48 (50.5%)	47 (49.5%)	
Age at inclusion (years)	34.4 ± 13.3	33.9 ± 14.2	35.0 ± 13.2	*p* = 0.659
Sex				
Female	43 (45.3%)	20 (46.5%)	23 (53.5%)	
Male	52 (54.7%)	28 (53.8%)	24 (46.2%)	
Follow-up (years)	8.53 ± 4.91	8.84 ± 4.88	8.22 ± 4.98	*p* = 0.522
Grading				
C1	5	0	5	
C2	51	14	37	
C3	39	34	5	
Pathological Fracture	12	11	1	
Tumour size	5.46 ± 2.71	5.14 ± 2.51	5.77 ± 2.88	*p* = 0.258
Neoadjuvant therapy				
Denosumab	20	13	7	
None	75	35	40	
Anatomical location				
Distal femur	23	12	11	
Proximal tibia	22	6	16	
Forearm	18	11	7	
Foot	10	5	5	
Proximal femur	6	4	2	
Hand	5	3	2	
Fibula	4	3	1	
Other	7	4	3	

**Table 2:** Potential risk factors and incidence of local recurrence.

Risk Factor	Local Recurrence Incidence Exposed Group/Number at Risk	Local Recurrence Incidence Unexposed Group/Number at Risk	Odds Ratio	95% CI	*p* Value
Female gender	8/43	11/52	0.85	0.30–2.35	0.757
Curettage	14/47	5/48	3.64	1.19–11.15	0.023
Denosumab	3/20	16/75	0.65	0.16–2.50	0.531
Campanacci G3	10/39	9/56	1.80	0.65–4.95	0.254
Age < 30 years	7/40	12/55	0.89	0.31–2.52	0.833
Size > 5 cm	11/38	8/57	2.06	0.75–5.60	0.155

**Table 3:** Local recurrence incidence in dependence on anatomical location.

	Treatment	Resection Local Recurrence/Number at Risk	Curettage Local Recurrence/Number at Risk	Total Local Recurrence/Number at Risk	Odds Ratio	95% CI	*p* Value
Location							
Proximal tibia		2/6	6/16	8/22	3.22	1.09–9.48	0.033
Forearm		1/11	2/7	3/18	0.76	0.19–2.96	0.695
Foot and hand		1/8	1/7	2/15	0.69	0.11–2.77	0.486
Distal femur		0/12	2/11	2/23	0.30	0.06–1.45	0.136
Other		1/4	3/3	4/7	1.29	0.36–4.52	0.688

## Data Availability

The data that support the findings of this study are available from the corresponding author upon reasonable request.
